# How to Elucidate Consent-Free Research Use of Medical Data: A Case for “Health Data Literacy”

**DOI:** 10.2196/51350

**Published:** 2024-06-18

**Authors:** Gesine Richter, Michael Krawczak

**Affiliations:** 1 Institute of Experimental Medicine, Division of Biomedical Ethics, Kiel University, University Hospital Schleswig-Holstein, Kiel, Germany; 2 German Center for Lung Research (DZL), Airway Research Center North (ARCN), Borstel, Germany; 3 Institute of Medical Informatics und Statistics, Kiel University, University Hospital Schleswig-Holstein, Kiel, Germany

**Keywords:** health data literacy, informed consent, broad consent, data sharing, data collection, data donation, data linkage, personal health data

## Abstract

The extensive utilization of personal health data is one of the key success factors of modern medical research. Obtaining consent to the use of such data during clinical care, however, bears the risk of low and unequal approval rates and risk of consequent methodological problems in the scientific use of the data. In view of these shortcomings, and of the proven willingness of people to contribute to medical research by sharing personal health data, the paradigm of informed consent needs to be reconsidered. The European General Data Protection Regulation gives the European member states considerable leeway with regard to permitting the research use of health data without consent. Following this approach would however require alternative offers of information that compensate for the lack of direct communication with experts during medical care. We therefore introduce the concept of “health data literacy,” defined as the capacity to find, understand, and evaluate information about the risks and benefits of the research use of personal health data and to act accordingly. Specifically, health data literacy includes basic knowledge about the goals and methods of data-rich medical research and about the possibilities and limits of data protection. Although the responsibility for developing the necessary resources lies primarily with those directly involved in data-rich medical research, improving health data literacy should ultimately be of concern to everyone interested in the success of this type of research.

## Data-Rich Research, Broad Consent, and Informedness

Various initiatives around the world are currently working on the technical and organizational requirements to make data from different sources and contexts usable for medical research (eg, MyHealthRecord in Australia, FINDATA in Finland, and the Medical Informatics Initiative in Germany). The starting points of these endeavors often are local, regional, or national health care data repositories that must nevertheless be highly linkable to allow full exploitation of their scientific value. This connectivity requirement implies that the data cannot be fully anonymized before being moved into the research domain.

One of the ethical prerequisites for research on humans—and thus for research using identifiable personal health data—is the informed consent of the data subjects. However, being properly informed requires that those affected (1) are capable of making self-determined decisions in the first place; (2) were informed about the nature, benefits, and risks of the research in question; (3) have understood the importance of this information; and (4) are able to decide voluntarily and without coercion for or against participation.

Not least because of the increasing relevance of hypotheses-free research approaches (keyword: big data), the storage and use of data for future, currently undeterminable purposes also play an increasingly important role in medical research. Recent studies have shown that patients and members of the general public are very willing to share personal health data for research (eg, [1]), even if no information about the purposes and aims of the research can be provided at the time consent is given. Notably, this attitude turned out to be mainly motivated by altruism, solidarity, and the idea of reciprocity. Since the paradigm of project-related informed consent is difficult to transfer to such unspecific practice, the World Medical Association changed its regulations on research with identifiable data when revising the Declaration of Helsinki in 2013 [2]. There was no longer a requirement for specific information about the subjects of future research, thereby paving the way for a new form of “broad consent.”

In essence, “broad consent” means the one-off, unspecific agreement to the use of one’s personal data for medical research without knowing who will access the data when and to what end. However, since the data in question are usually collected in a clinical care context, the suitability and practicality of broad consent as a legitimation for their research use is limited. First, the temporal and spatial linking of the consent process to care measures can lead to incorrect therapeutic [3] and diagnostic [4] assumptions on the side of the patient. Second, in the time available, it is hardly possible to create sufficient understanding of the benefits and risks of the envisaged research, despite great efforts to ensure that the corresponding information and consent documents are legible. Finally, asking for consent during clinical care bears a substantial risk of low and unequal approval rates, which can lead to methodological problems in the scientific use of the data.

In view of these shortcomings, and of the proven willingness of people to contribute to medical research by sharing personal health data, the means to achieve practically feasible and truly informed consent needs to be reconsidered. In particular, is consent-free data use for medical research, combined with the possibility of straightforward opt-out by the data subjects after thorough consideration, a better option for legitimizing the secondary use of health data? This question is all the more justified as numerous studies in the United Kingdom, Iceland, Norway, Sweden, and Germany, among others, have shown a generally positive attitude of people toward such a regulation (eg, United Kingdom [5]; United Kingdom, Iceland, Norway, and Sweden [1]; Norway [6]; and Germany [7,8]).

In the following, we will first introduce “data donation” as an opt-out approach to legitimizing the secondary research use of personal medical data. Since opt-out would imply that patients are no longer informed directly about the research-associated risks and benefits, alternative ways of information provision must be explored in the context of data donation if the paradigm of informedness was to be maintained. We therefore also introduce the concept of “health data literacy,” defined as the capacity to find, understand, and evaluate information about data-rich medical research. Although a case for general health data literacy can be made independently of the issue of patient consent, its consideration becomes particularly urgent for the latter if the framework of consenting was to change from opt-in to opt-out.

## Data Donation: Consent-Free Research Use of Medical Data Plus Opt-Out

The European General Data Protection Regulation (EU-GDPR) gives European member states considerable leeway with regard to permitting the research use of health data without consent. While Article 9 Paragraph 1 of the EU-GDPR clearly prohibits the processing of personal genetic, biometric, or health data, Article 9 Paragraph 2(j) explicitly exempts processing for scientific research purposes [9]. In addition, Article 89 allows national legislation to provide for this exception, subject to appropriate safeguards for the rights and freedom of the data subjects.

In Germany, the ethical, legal, technological, and organizational framework of the consent-free use of health data was examined in 2020 in a detailed report to the Federal Ministry of Health [10]. In addition to its legal admissibility, the report addressed the scientific benefits of such an approach, its impact upon the right of informational self-determination, and the necessity and possibilities for fair involvement of the data subjects. The authors concluded that it would be possible in Germany to replace the requirement for explicit consent for research with personal medical data by an equivalent legal permission, combined with an easy-to-exercise opt-out. Under certain conditions, such “data donation” (as it was termed in the report) would be both legally possible and ethically reasonable.

The above notwithstanding, the authors were also unequivocal that the actual process of data access by potential users should be independent of whether access is legitimized by opt-in or opt-out. The involvement of an ethics board or a use-and-access committee that reviews and decides data applications remains essential in both cases. Notably, such institutions also play an important role in weighing the potential risks and benefits of individual research projects, a legitimation mechanism that was deliberately placed on the same level as consent by the EU-GDPR.

Importantly with a view to the following considerations, the report clarified that, in addition to technical and organizational protective measures, one prerequisite for the acceptability of data donation would be that patients and citizens were sufficiently well informed about it. This proviso inevitably leads to the question of how sufficient knowledgeability can be achieved if the decision about sharing one’s data for research purposes is no longer made actively, following thorough verbal explanation, but passively by exercising or not exercising a right of objection.

## Limits of Top-Down “Informability”: the COVID-19 Infodemic as an Example

Since data donation, in the above sense, would be temporally and spatially decoupled from medical care and instead be anchored in everyday life, alternative offers of information would have to compensate for the lack of direct communication with medical or scientific experts [11]. Yet, the COVID-19 pandemic recently highlighted that the expansion of top-down media campaigns alone is not sufficient to adequately convey the complex aspects of medical research to the general public. Instead, it turned out that, despite the general increase in information provided, many people who opposed vaccination in the first place still were not sufficiently receptive to scientific facts [12]. Moreover, even some kind of social grouping occurred along people’s vaccination status, and the COSMO study carried out in Germany and Austria revealed that the stronger the identification with being unvaccinated, the lower the inclination to change this status, and the greater the feeling of discrimination [13]. Obviously, the ability to become informed (“informability”) had reached its limits in view of the amount of information available, a paradox that lamentably also had a negative impact upon the effectiveness of public health measures taken.

In connection with the COVID-19 pandemic, the World Health Organization (WHO) coined the term “infodemic” for the increasingly observed susceptibility of people to fake news as a result of reduced informability. According to the WHO, the infodemic caused a high degree of uncertainty in the population, a greater willingness to engage in health-damaging and risk-taking behavior, and an increased distrust of the health authorities [14]. The “Infodemic Management” called for by the WHO aimed to enable the population to better understand information from health experts and to become more resistant to misinformation [15].

## Ways to Better Informability?

In view of its complexity, it seems unrealistic to convey all relevant information about the research use of personal health data at once. We therefore propose “health data literacy” as a basis for better informability of the general population and, hence, as a means to uphold the paradigm of informed consent even in the context of data donation in the above sense. For a well-informed general public, data donation would indeed mean nothing more than a change in decision format—from opt-in to opt-out.

In a narrower sense, the word “literacy” stands for the ability to read and, thereby, to acquire education and knowledge. According to the Organisation for Economic Co-operation and Development (OECD), understanding and interpreting written material should enable citizens to develop their own potential and to fully participate in societal affairs [16]. The starting point of our considerations on health data literacy therefore will be a class of communication models that focus upon the possible causes of limited informability.

One decisive factor for the success of communication is the thought system of the recipient. Since we often have little time to consider large amounts of everyday information, we believe statements that we have heard very often to be more credible than others [17]. This effect is reinforced by the phenomenon of group polarization: those who share a widespread opinion on complex issues are more likely to be reserved about new information and tend to believe whatever confirms their own viewpoint rather than information that does not fit. This selective form of information intake can, for example, increase polarization in social disputes even in the presence of reliable evidence and information [18]. The concept of health data literacy picks up on the basic idea of these communication models and aims to create anchor points in the knowledge base of people, where information on the benefits and risks of data-rich medical research can be stored and evaluated.

Value congruence approaches aim in a similar direction, in that they try to increase trust in certain institutions [19,20]. Such trust will be greater when more individuals perceive that their interests and values are shared by the institution in question, because trust is also largely based upon the perception of common values. This applies all the more to institutions that use health data for research, and it is therefore in the best interest of such institutions to develop and represent values that are highly rated by the public [19,20]. In this context, widespread health data literacy could form the breeding ground for the perception of a congruence of values and, thus, for greater trust in the recipients and beneficiaries of data donation.

## The Concept of “Health Data Literacy”

An individual’s health data literacy is positioned between their health literacy and their data literacy, where the latter in particular has been promoted politically, for example, by the data strategy of the German federal government [21].

- In view of the increasingly specific treatment options promised by so-called “precision medicine,” citizens would be well advised to take an interest in issues related to disease prevention and medical care [22]. The associated term “health literacy” summarizes both the motivation and the ability to find, understand, evaluate, and apply the information underlying personal health–related decisions [23]. Numerous international studies have measured and compared the level of health literacy in different populations (eg, [24]), as well as spurring considerations as to how health literacy can be increased (eg, [25]).
- The term “data literacy” refers to knowledge about data and their use in general, including legal, ethical, and social aspects. Data literacy thus forms the basis of personal self-determination in an increasingly digitalized society [26]. The aim of data literacy is an ability to weigh one’s own personal rights against the potential benefits of making personal data available to others [27].

In combining both abovementioned terms, “health data literacy” stands for the capacity to find, understand, and evaluate information about the risks and benefits of medical research with personal health data; to compare this information with one’s own values; and to act accordingly. Health data literacy is thus a transformer of information into informed action, aimed at a level of thematic familiarity that enables self-determined decision-making about the sharing of one’s own health data with the research community. Specifically, health data literacy should at the very least include basic knowledge about the goals and methods of data-rich medical research and about the possibilities and limits of data protection.

**Figure 1.**
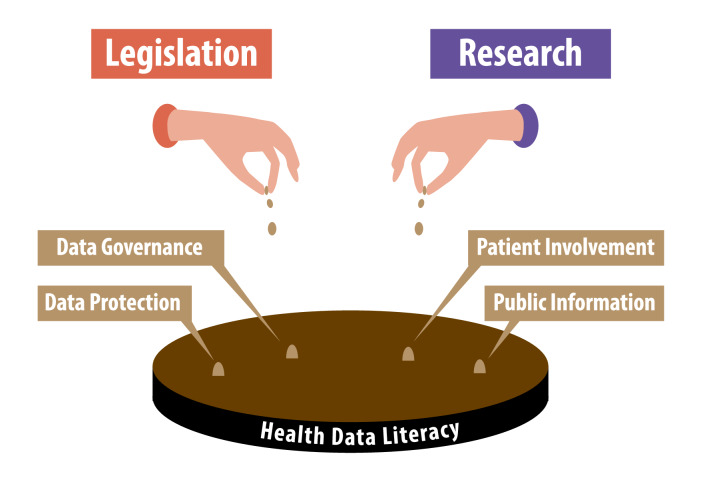
Health data literacy as a breeding ground for the societal acceptance of data donation.

The increasing relevance of personal health data for medical research has led to a large number of measures to increase the societal acceptance of the use of such data. However, legislative regulations on data governance and data protection, as well as efforts to increase patient involvement and public information, are likely to have greater impact when they are met with more adequate prior knowledge in the sense of health data literacy (Figure 1).

**Figure 2.**
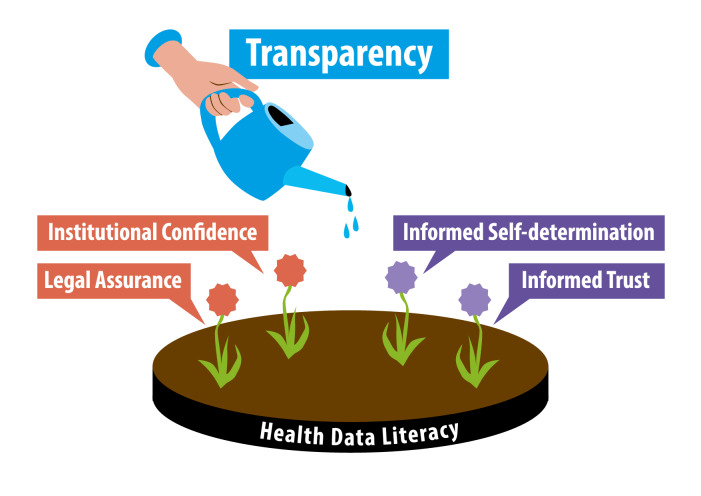
Transparency nourishes confidence and trust in data-rich medical research.

When the mental anchor points set by health data literacy receive information on scientific successes, new technical and organizational developments, as well as possible setbacks of data-rich medical research (keyword: transparency), this information can be evaluated competently by the recipient and compared to their own expectations. In the aftermath of such reflections, informed self-determination and sufficient trust in regulations and institutions can develop (Figure 2).

## Outlook: Feasibility and Implementation of Health Data Literacy

Numerous international studies among patients and in the general population have revealed a broad positive attitude toward the provision of personal health data to medical research (eg, [28]). This approval was consistently found to be driven by a sense of reciprocity, that is, a wish to give something back after benefiting from research (eg, [29,30]). Evidence also emerged for the widespread belief in a social duty of citizens to contribute their own data to research, independent of their personal benefit [31,32]. At the same time, however, a craving for more detailed information was observed, up to and including the view that every individual is responsible themselves to find out about the nature and benefits of research with personal health data (eg, [33]).

In summary, we are thus in a situation where (1) there is little doubt about the need to utilize personal health data from different contexts to achieve the goals of modern medical research, (2) the consent-free use of such data meets broad approval by the general public, and (3) there is a widespread willingness of people to acquire the knowledge necessary to make a self-determined decision about data donation. The most compelling argument for general health data literacy is therefore self-evident: widespread background knowledge of the risks and benefits of data-rich medical research would allow the paradigm of informedness to be maintained even if consent to participation in research is implement by opt-out, rather than opt-in.

However, the appeal of general health data literacy undoubtedly goes beyond the issue of data donation. Its necessity arises from the increasing complexity of data-rich medical research, which can no longer be explained adequately via waiting room leaflets or doctor consultations. We are also aware that improved health data literacy could, in principle, help to reduce some of the misunderstandings of patients that we somehow held against broad consent when advocating data donation. However, in view of the many advantages of data donation summarized above, we think that only little importance should be attached to this possibility.

Attempts to establish general health data literacy should strive for a certain level of competence across as broad a proportion of the population as possible. This goal not only expresses fairness and ensures equal representation of different societal groups in medical research but can also help to reduce the vulnerability to fake information as a potential threat to public health, as observed during the COVID-19 pandemic. Achieving equity in practice will require the development and provision of target group–specific offers of information and education. One particularly efficient way to increase health data literacy across the board would be to start this process in school, as suggested previously to strengthen health literacy [25]. This approach is not only easy to implement in practice; it would also offer the opportunity to use children as multipliers among friends and family.

Further research is needed to determine exactly what kind of information should be communicated, in what form, and to whom to improve health data literacy in a given population. These questions are ideally answered through cocreation research involving representatives of different target groups to enhance the credibility of the education curriculum and content among end users. However, although the responsibility for developing the necessary resources lies primarily with those directly involved in data-rich medical research, improving health data literacy should ultimately be of concern to everyone interested in the success of this type of research.

## Abbreviations

EU-GDPR: European General Data Protection Regulation
OECD: Organisation for Economic Co-operation and Development
WHO: World Health Organization
